# Associations between serum trace elements and the risk of nasopharyngeal carcinoma: a multi-center case-control study in Guangdong Province, southern China

**DOI:** 10.3389/fnut.2023.1142861

**Published:** 2023-07-03

**Authors:** Xin-Yu Ge, Shang-Hang Xie, Hao Wang, Xin Ye, Wenjie Chen, Hang-Ning Zhou, Xueqi Li, Ai-Hua Lin, Su-Mei Cao

**Affiliations:** ^1^Department of Cancer Prevention Center, Sun Yat-sen University Cancer Center, Guangzhou, China; ^2^School of Public Health, Sun Yat-sen University, Guangzhou, China; ^3^Department of Colorectal Surgery, State Key Laboratory of Oncology in South China, Collaborative Innovation Center for Cancer Medicine, Sun Yat-sen University Cancer Center, Guangzhou, China; ^4^State Key Laboratory of Oncology in South China, Collaborative Innovation Center for Cancer Medicine, Guangdong Key Laboratory of Nasopharyngeal Carcinoma Diagnosis and Therapy, Guangzhou, China

**Keywords:** cadmium (Cd), manganese (Mn), nasopharyngeal carcinoma (NPC), head and neck cancer, logistic regression, restricted cubic splines, odds ratio, trace element (TE)

## Abstract

**Background:**

Associations between trace elements and nasopharyngeal carcinoma (NPC) have been speculated but not thoroughly examined.

**Methods:**

This study registered a total of 225 newly diagnosed patients with NPC and 225 healthy controls matched by sex and age from three municipal hospitals in Guangdong Province, southern China between 2011 and 2015. Information was collected by questionnaire on the demographic characteristics and other possibly confounding lifestyle factors. Eight trace elements and the level of Epstein–Barr virus (EBV) antibody were measured in casual (spot) serum specimens by inductively coupled plasma–mass spectrometry (ICP-MS) and enzyme-linked immunosorbent assay (ELISA), respectively. Restricted cubic splines and conditional logistic regression were applied to assess the relationship between trace elements and NPC risk through single-and multiple-elements models.

**Results:**

Serum levels of chromium (Cr), cobalt (Co), nickel (Ni), arsenic (As), strontium (Sr) and molybdenum (Mo) were not associated with NPC risk. Manganese (Mn) and cadmium (Cd) were positively associated with NPC risk in both single-and multiple-element models, with ORs of the highest tertile compared with the reference categories 3.90 (95% CI, 1.27 to 7.34) for Mn and 2.30 (95% CI, 1.26 to 3.38) for Cd. Restricted cubic splines showed that there was a linear increasing trend between Mn and NPC risk, while for Cd there was a J-type correlation.

**Conclusion:**

Serum levels of Cd and Mn was positively related with NPC risk. Prospective researches on the associations of the two trace elements with NPC ought to be taken into account within the future.

## 1. Introduction

Nasopharyngeal carcinoma (NPC) is a malignant epithelial tumor arising from the nasopharyngeal mucosal lining (1) and has a high incidence in southern China (2). 133,354 new cases of NPC were diagnosed in 2020 around the world, and around half of the patients are from southern China (3). Epstein–Barr virus (EBV) infection is a major risk factor for undifferentiated NPC, which is responsible for over 90% of the total patients in the areas with high incidence (4). EBV is a ubiquitous herpesvirus that is carried by more than 90% of people worldwide, but only a small division suffer from NPC (5), which could not explain the unique regional characteristics of NPC. Therefore, cofactors must mediate the effect of EBV on NPC.

Trace elements are widely present in the environment and can influence the human body complexly by accumulating through water or contaminated food intake. Although in the normal range some trace elements may be essential in metabolism, they can become potentially toxic and produce negative effects on health at higher concentrations or a long-term exposure (6). This may be due to the ability of trace elements to increase oxidative stress or change the repair of DNA damage (7, 8). Several studies have reported that certain trace elements are closely related to the regional endemic tumors. For example, the regional high prevalence of liver cancer in Taiwan and lung cancer in the miners of Gejiu, Yunnan Province are associated with excessive exposure of arsenic (As) in water and Tin in mine dust, respectively (9, 10).

As a regional endemic tumor, the relationship between trace elements and NPC have been doubted for several decades (11). Several ecological studies have shown that the concentrations of As and cadmium (Cd) in rice and some plant-based food in Guangdong exceeded the food safety threshold (12). In addition, the concentrations of nickel (Ni) in rice, drinking water or local residents’ hair are significantly higher in high-risk regions than those in low counterparts (12–14). Epidemiological surveys have reported that trace elements Cd and zinc were found to be positively associated with NPC, while for strontium (Sr), calcium and magnesium were negatively associated (15). However, previous studies have not provided convincing evidence for the impact of trace elements on NPC, in part because those were mainly ecological studies, or limited by their small sample sizes, or focused on individual trace element, or in the occupational populations (16–18). A well-designed case-control study is still required to explore the associations of commonly exposed trace elements with NPC risk in the general population of endemic regions.

In the present study, a case–control study in three municipal hospitals in NPC-endemic southern Chinese province was conducted to explore the serum concentrations of eight trace elements (chromium, Cr; manganese, Mn; cobalt, Co; Ni; As; Sr; molybdenum, Mo and Cd) with NPC risks, with the possible dose–response relationships and the interactions between some trace elements and other potential risk factors on NPC risks were also explored.

## 2. Materials and methods

### 2.1. Study population

Two hundred and forty newly diagnosed NPC cases were selected consecutively in three municipal hospitals in Guangdong Province, southern China from 2011 to 2015 years. 15 cases were excluded, including 4 samples collected after treatment, 5 cases with insufficient serum volume, and 6 cases with incomplete information, leaving 225 eligible cases for this study. The control group was recruited from the health check-ups of the same hospital in the same period and was randomly matched to cases at 1:1 ratio by age (±5 years old), sex and city of residence. The Institutional Research Ethics Committee of Sun Yat-sen University Cancer Center (SYSUCC) approved this study.

### 2.2. Sample collection

After fasting for 12 h, 4 mL of venous blood were collected from the subjects in an inert separation gel coagulant tube in the morning. Samples were separated and packed within 12 h to obtain serological samples, and immediately transferred to the laboratory for storage in a refrigerator at −80°C until assay.

Interviewers were trained and an electronic organized questionnaire was utilized by interviewers to face-to-face interviews. To minimize interviewer bias, each questioner was assigned a roughly same number of cases and controls. The collected information contains the following factors: demographic characteristics; current occupation; education levels; residential history; chronic sinusitis history; family history of NPC; cigarette smoking and alcohol consumption.

### 2.3. Analytical procedures

Serum concentrations of the eight concerned trace elements (i.e., Cr, Mn, Co, Ni, As, Sr., Mo, and Cd) were determined by using inductively coupled plasma–mass spectrometry (ICP-MS, Thermo Fisher Scientific company, Germany). The detection limits of each element were demonstrated (Supplementary Table S1). In brief, each 200 μL of serum samples was weighted into a 5 mL pressure vessel, adding 1 ml 65% HNO_3_ and 1 ml 30% H_2_O_2_. Three parallel tests were carried out for each sample, and the average value of the three tests was taken. Samples were treated as a half value of the lower detection limit when they below the detection limit and were excluded when they higher than or lower than three standard deviations of the control group (log-transformed).

The serum samples were detected blindly by the inorganic and element analysis platform in the Test Center of Sun Yat-sen University. 5% serum samples were randomly selected for retest, and Intra-class Correlation Coefficients (ICC) was calculated to assess the test–retest reliability. Supplementary Table S2 shows that the reliability is good, with most of the trace elements having an ICC greater than 0.7. The detection limit of specific elements and the calibration equation show a good linear relationship between the concentration of each element and the signal strength of the instrument (all of R^2^ > 0.999; data not shown).

Viral capsid antigen to IgA (VCA-lgA) was measured by using the commercial enzyme-linked immunosorbent assay (ELISA) kits (EUROIMMUNAG, Lübeck, Germany) at the central laboratories of SYSUCC. Our previous study has described the detailed procedure of the EBV serological test (19). In our study, according to the median of antibody level of all subjects, the group with higher levels of VCA-lgA was ≥1.212.

### 2.4. Statistical analysis

The characteristics between cases and controls were compared by Chi-square tests or Mann–Whitney *U* tests. And the relationships between each trace elements were evaluated by using Spearman’s rank correlation. We used restricted cubic splines to explore nonlinear relationships between trace elements (log-transformed) and the NPC risk (OR). Knots within the splines were set at 5th, 35th, 65th and 95th percentile, respectively. Conditional logistic regression models with single and multiple-element models were applied to estimate the associations between each trace element and NPC. The concentration of trace elements related with the lowest NPC risk was the concentration with the lowest OR on the spline curve. Considering that the lowest OR of nonlinear trace elements is located in the second tertile of the serum trace element concentration of the controls, the associations between three predefined trace elements concentrations categories and NPC risks were examined: three equally distributed categories of trace elements concentration of controls were defined by the 33rd and 67th centiles. The reference criterion for these studies was the trace elements level associated with the lowest NPC risk. Adjusted factors included gender (male, female), age (years), chronic rhinitis status (yes, no), first-degree family history of NPC (yes, no), drinking (ever, never), smoking status (ever, never) and the levels of VCA-lgA (higher, lower). Ever smokers were defined as those who reported having smoked more than 1 cigarette every 1–3 days for 6 consecutive months, and ever drinkers were defined as those who consumed more than 1 glass of alcoholic beverage (including wine, beer and liquor) per day for an equal period. Respondents who had quit smoking or alcohol for less than 1 year were considered ‘current’ in our study; Those who had quit for longer were considered “former.” Both “former” and “current” consumers were collectively called “ever” consumers (20, 21). Odds ratios (ORs) and 95% confidence intervals (95% CIs) were calculated to evaluate NPC risk. For linear trace elements, *p*-values for the trend were treated by the median of each element tertiles as continuous variables.

We used likelihood ratio tests to compare interaction term between each serum trace element and potential modifier in logistic regression model. Stratification analysis by smoking and the levels of EBV antibody was applied to estimate the relationship between the selected serum trace element and NPC risk. In the multiple-elements model, we used the Bonferroni correction to make multiple comparisons, and values of *p* < 0.01 were considered statistically significant. For other analyses, a two-tailed *p* < 0.05 was considered statistically significant. Data were analyzed by R statistical packages (The R Foundation; http://www.r-project.org; version 4.1.1).

## 3. Results

### 3.1. Characteristics of study population

Table 1 displayed the distribution of demographic variables and possible risk factors for NPC among the 225 NPC patients and 225 controls. NPC patients, compared with controls, were more likely to have chronic rhinitis, to have cigarette smoking, to have consumed alcohol, to have higher EBV antibody levels and to have a first-degree NPC family history. The mean age was 50.00 ± 11.03 years old in NPC patients and 48.91 ± 11.93(SD) years old in control group, accordingly (*p* > 0.05). In addition, 70.22% of patients with NPC were male (*N* = 158). There was no statistical difference in occupational types and education levels between the case and control groups.

**Table 1:** Characteristics of nasopharyngeal carcinoma (NPC) cases and controls.

Characteristics	Cases (*N* = 225)	Controls (*N* = 225)	*P-*value
Age (years, Mean ± standard deviation)	50.00 ± 11.03	48.91 ± 11.93	0.491
Residential area, *n* (%)			0.970
Zhongshan	59 (26.22)	57 (25.33)	
Sihui	56 (24.89)	58 (25.78)	
Zhaoqing	110 (48.89)	110 (48.89)	
Gender, *n* (%)			0.363
Male	158 (70.22)	148 (65.78)	
Female	67 (29.78)	77 (34.22)	
Chronic rhinitis status, *n* (%)			<0.001
No	192 (85.33)	213 (94.67)	
Yes	33 (14.67)	12 (5.33)	
Cigarette smoking, *n* (%)			0.072
Never	95 (42.22)	115 (51.11)	
Ever	130 (57.78)	110 (48.89)	
Alcohol consumption, *n* (%)			0.006
Never	142 (63.11)	170 (75.56)	
Ever	83 (36.89)	55 (24.44)	
First-degree family history of NPC, *n* (%)			<0.001
No	202 (89.78)	220 (97.78)	
Yes	23 (10.22)	5 (2.22)	
EBV antibodies (VCA-lgA), *n* (%)			<0.001
Lower level	27 (12.00)	198 (88.00)	
Higher level	198 (88.00)	27 (12.00)	
Education levels, *n* (%)			0.977
None or primary school	91 (40.44)	87 (38.67)	
Secondary school	89 (39.56)	90 (40.00)	
High school	36 (16.00)	38 (16.89)	
University or more	9 (4.00)	10 (4.44)	
Current occupation, *n* (%)			0.271
Unemployed	8 (3.56)	7 (3.11)	
Farmer	103 (45.78)	100 (44.44)	
Blue collar	65 (28.89)	69 (30.67)	
White collar	24 (10.67)	25 (11.11)	
Other/unknown	25 (11.11)	24 (10.67)	

Student’s *t*-tests and Chi-square tests were used to estimate differences of variables between the case and control group according to their distributions.

### 3.2. Concentration levels of the trace elements in the case and control group

A total of eight trace elements in serum samples were tested between cases and controls. The detection limits of each element were displayed in Supplementary Table S2. Except Sr., the levels of other trace elements between NPC patients and controls were significantly distinct (*p* < 0.05). The median concentrations of five elements were higher in the NPC patients than in the control group, including Co (0.66 vs. 0.32 μg/L), Ni (4.17 vs. 1.77 μg/L), Cr (2.99 vs. 2.15 μg/L), Mn (12.93 vs. 4.52 μg/L) and Cd (0.91 vs. 0.20 μg/L), whereas As (1.99 vs. 2.59 μg/L) and Mo (0.79 vs. 1.14 μg/L) values were lower in the cases (Table 2). Most of the elements showed significant correlations (Supplementary Table S3), with the highest Spearman’s rank correlation 0.720 between Mn and Co.

**Table 2:** Concentrations of trace elements in serum of controls and cases (*N* = 450; μg/L).

Elements	Controls (*N* = 225)				Cases (*N* = 225)				*P*-value
	Mean (SD)	Percentile 25	Median	Percentile 75	Mean (SD)	Percentile 25	Median	Percentile 75	
Cr	3.28 (4.01)	0.93	2.15	3.92	4.33 (4.39)	1.58	2.99	6.05	<0.001
Mn	7.06 (7.70)	2.11	4.52	9.59	15.12 (10.10)	7.38	12.93	21.96	<0.001
Co	0.43 (0.35)	0.17	0.32	0.53	0.68 (0.41)	0.38	0.66	0.92	<0.001
Ni	5.24 (10.45)	0.50	1.77	4.37	6.39 (8.08)	0.92	4.17	8.94	<0.001
As	5.55 (7.00)	1.86	2.59	5.22	5.46 (8.05)	1.42	1.99	4.24	<0.001
Sr	30.34 (10.12)	23.13	28.88	35.26	31.55 (12.74)	23.20	29.13	38.15	0.519
Mo	1.62 (1.87)	0.13	1.14	2.14	1.13 (1.49)	0.05	0.79	1.63	0.004
Cd	0.42 (0.71)	0.06	0.20	0.58	1.82 (2.11)	0.05	0.91	3.21	<0.001

Cr, Chromium; Mn, Manganese; Co, Cobalt; Ni, Nickel; As, Arsenic; Sr, Strontium; Mo, Molybdenum; Cd, Cadmium. *P*-values are used for Mann–Whitney *U*-tests for continuous variables according to the distribution.

### 3.3. Association between serum trace elements and the risk of nasopharyngeal carcinoma

**Figure 1:**
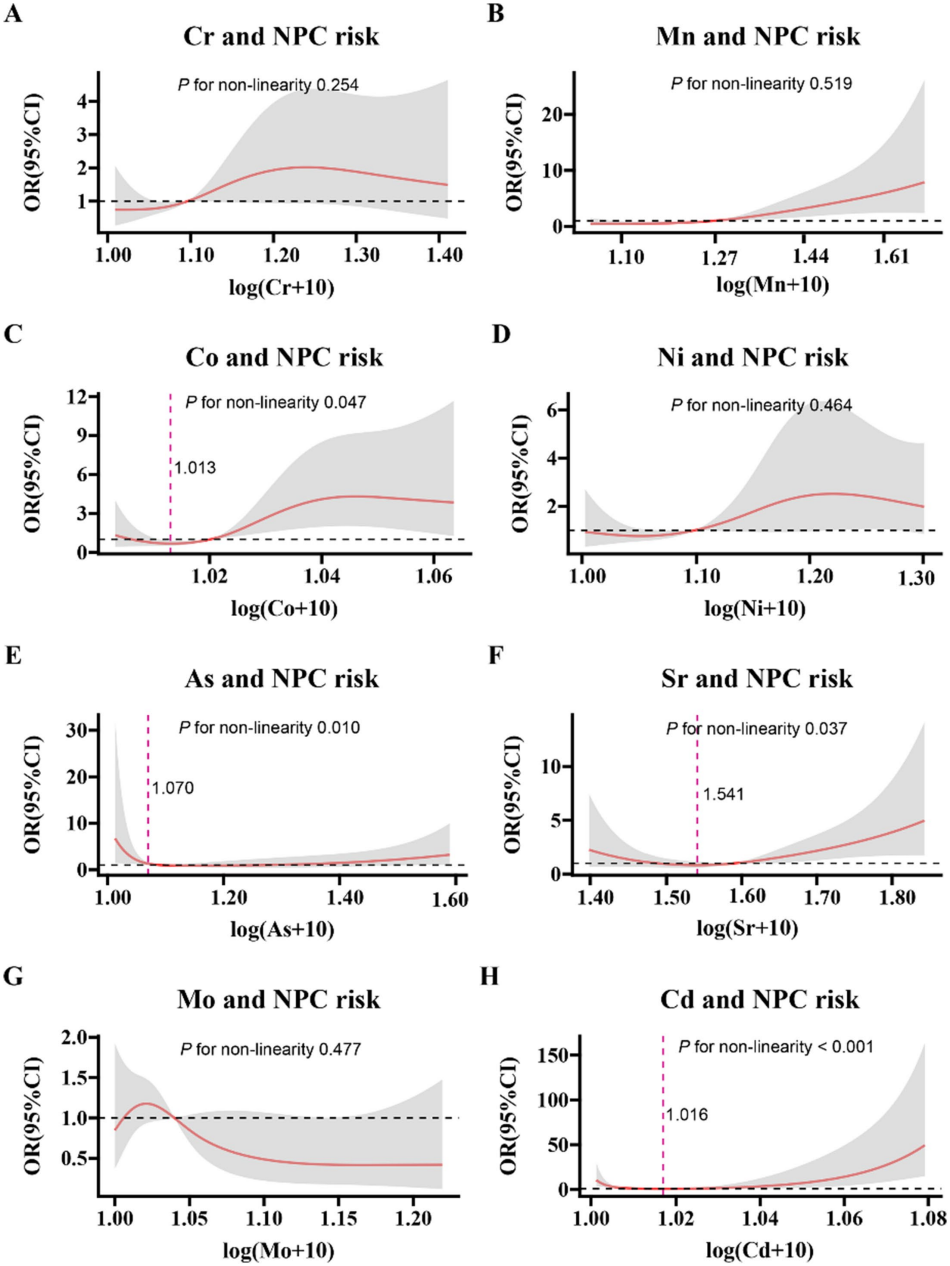
Nonlinear associations between trace elements in serum (log-transformed) and the risk of NPC (Odds Ratio) in the single-element models. Nonlinear associations were presented by the restricted cubic splines, and adjusted by age (years), gender (male, female), chronic rhinitis status (yes, no), first-degree family history of NPC (yes, no), drinking (ever, never), smoking status (ever, never) and the levels of VCA-lgA (higher, lower). Shading in the plots indicated the confidence interval (95%CI). The knots in the plots were set at 5th, 35th, 65th, and 95th percentile, respectively. Dashed red lines indicate the concentration of serum trace elements with the lowest risk of NPC for nonlinear trace element. Figure A-H represented the risk of NPC with the concentration of serum chromium, manganese, cobalt, nickel, arsenic, strontium, molybdenum and cadmium, respectively.

**Figure 2:**
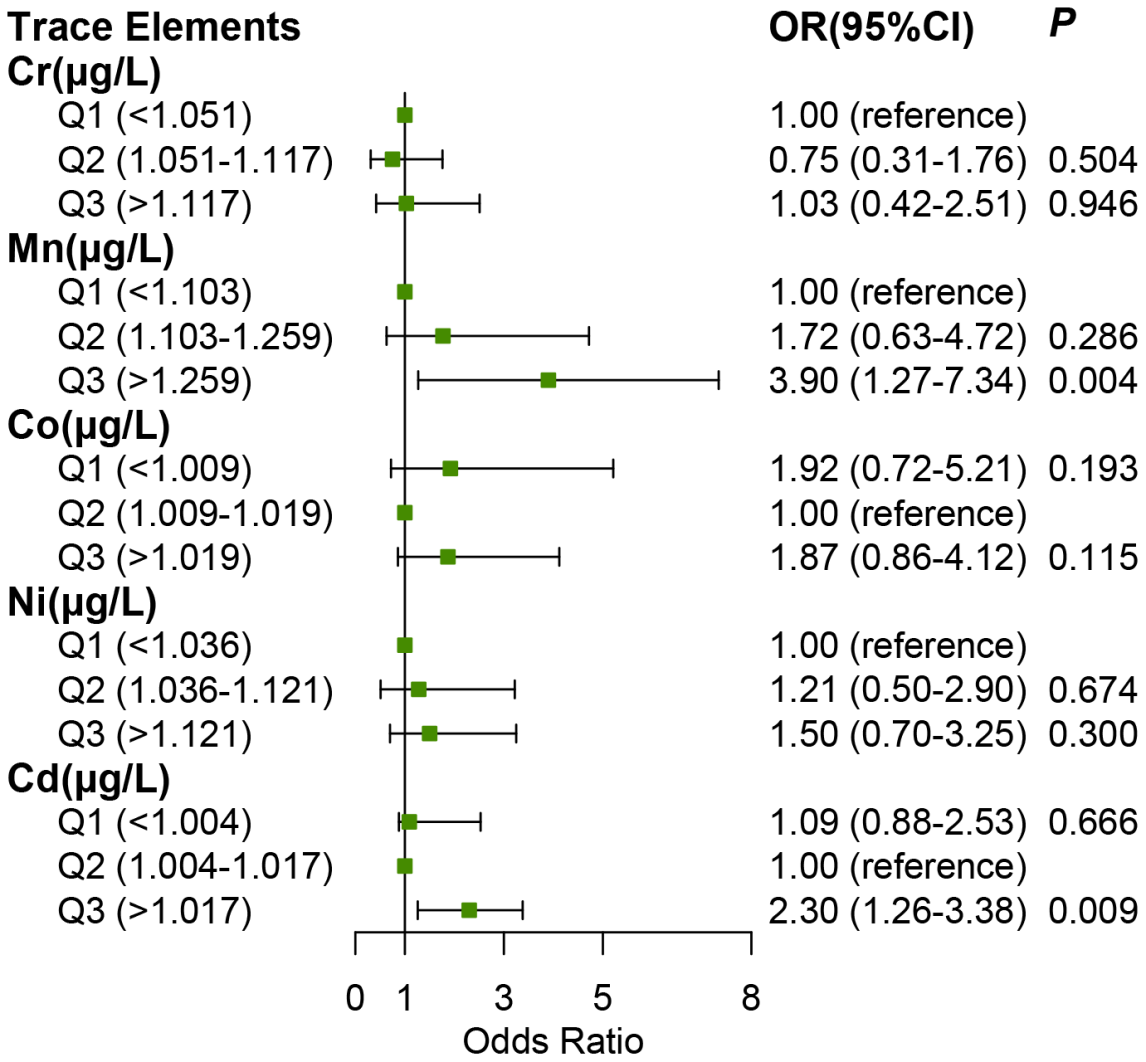
Associations between trace elements in serum (log-transformed) and the risk of NPC (Odds Ratio) in the multiple-element models. Adjusted by age, gender, chronic rhinitis status, smoking, drinking, first-degree family history of NPC and VCA-lgA. OR: odds ratio. CI: confidence interval. Trace elements were added 10 and log transformed.

We used restricted cubic splines to flexibly model and visualize the relation of serum trace elements (log-transformed) with NPC risk (Figure 1). After adjusting for potential confounders, the restricted cubic spline showed U-shaped correlations between Co and Sr concentrations on a continuous scale and NPC risk, J-shaped associations for Cd and As and linear relationships for Cr, Mn, Ni, and Mo. The adjusted ORs for the risk of NPC related with linear and nonlinear serum trace elements in the single-element models were shown in Tables 3, 4, respectively. After adjusting different potential confounders, no significant associations were found between serum As, Sr. and Mo and the risk of NPC. Serum Cr, Mn, Co, Ni and Cd were positively associated with the risk of NPC, with the OR of the highest tertile of trace elements vs. the reference category 3.90 (95%CI, 1.27 to 7.34) for Mn and 2.30 (95%CI, 1.26 to 3.38) for Cd (Figure 2) after further adjusting the five metals in the multiple-elements models. The variance inflation factor (VIF) was <1.5 for all the five trace elements indicating collinearity was not a concern.

**Table 3:** Odds ratios for the association between serum levels of trace element (Linear) and the risk of nasopharyngeal carcinoma based on the single-element model (95% confidence intervals).

Serum trace elements (μg/L)		Tertiles of serum trace elements (μg/L)			*P*-trend^*^
		Q1	Q2	Q3	
**Cr**					
	N (controls/cases)	148 (74/74)	156 (74/82)	146 (77/69)	
	Model 1	Reference	1.30 (0.78–2.19)	2.21 (1.36–3.62)	0.012
	Model 2	Reference	1.31 (0.78–2.19)	2.27 (1.39–3.72)	0.009
	Model 3	Reference	1.17 (0.54–2.54)	2.38 (1.14–5.06)	0.048
**Mn**					
	N (controls/cases)	92 (74/18)	116 (74/42)	242 (77/165)	
	Model 1	Reference	2.52 (1.32–4.95)	9.54 (5.22–12.25)	<0.001
	Model 2	Reference	2.63 (1.38–5.19)	9.90 (5.40–12.98)	<0.001
	Model 3	Reference	1.72 (0.70–4.29)	5.13 (2.23–8.16)	<0.001
**Ni**					
	N (controls/cases)	129 (74/55)	119 (74/45)	202 (77/125)	
	Model 1	Reference	0.72 (0.42–1.23)	1.99 (1.25–3.17)	<0.001
	Model 2	Reference	0.69 (0.40–1.18)	1.99 (1.25–3.18)	<0.001
	Model 3	Reference	1.21 (0.55–2.71)	2.22 (1.11–4.49)	0.015
**Mo**					
	N (controls/cases)	138 (74/64)	190 (74/116)	122 (77/45)	
	Model 1	Reference	0.78 (0.50–1.23)	0.40 (0.24–0.66)	0.006
	Model 2	Reference	0.79 (0.50–1.25)	0.40 (0.24–0.66)	0.007
	Model 3	Reference	0.96 (0.48–1.92)	0.61 (0.44–1.03)	0.105

* P-trend was estimated from the conditional logistic regression models according to the median value of each quartile of elements as a continuous variable.

Model 1 Adjusted for age, gender. Chronic rhinitis status, drinking and first-degree family history of NPC. Model 2 Further adjusted by smoking status (never/ever). Model 3 Further adjusted by the levels of VCA-lgA (lower/higher). Trace elements were added 10 and transformed by log.

**Table 4:** Odds ratios for the association between serum levels of trace element (nonlinear) and the risk of nasopharyngeal carcinoma based on the single-element model (95% confidence intervals).

Serum trace elements		N controls/cases	Model 1		Model 2		Model 3	
			OR (95%CI)	*P*	OR (95%CI)	*P*	OR (95%CI)	*P*
**Co**								
	Q1 (<1.009)	100 (72/28)	0.70 (0.38–1.29)	0.261	0.70 (0.38–1.30)	0.263	1.07 (0.45–2.56)	0.880
	Q2 (1.009–1.019)	115 (76/39)	Reference		Reference		Reference	
	Q3 (>1.019)	225 (77/158)	3.96 (2.43–6.57)	<0.001	3.99 (2.45–6.63)	<0.001	2.88 (1.43–5.91)	0.003
**As**								
	Q1 (<1.060)	146 (73/73)	2.69 (1.63–4.49)	<0.001	2.76 (1.67–4.63)	<0.001	1.91 (0.91–4.06)	0.087
	Q2 (1.060–1.133)	162 (74/88)	Reference		Reference		Reference	
	Q3 (>1.133)	142 (78/64)	1.19 (0.75–1.90)	0.458	1.20 (0.76–1.92)	0.434	1.06 (0.53–2.10)	0.869
**Sr**								
	Q1 (<1.538)	141 (74/67)	1.15 (0.70–1.88)	0.587	1.14 (0.70–1.88)	0.590	0.98 (0.46–2.07)	0.964
	Q2 (1.538–1.624)	141 (74/67)	Reference		Reference		Reference	
	Q3 (>1.624)	168 (77/91)	1.47 (0.91–2.36)	0.113	1.46 (0.91–2.36)	0.115	2.29 (1.10–4.90)	0.028
**Cd**								
	Q1 (<1.004)	154 (74/80)	2.39 (1.35–4.29)	0.003	2.46 (1.38–4.44)	0.003	1.99 (0.87–4.59)	0.105
	Q2 (1.004–1.017)	150 (74/76)	Reference		Reference		Reference	
	Q3 (>1.017)	146 (77/69)	3.61 (2.28–5.79)	<0.001	3.61 (2.28–5.78)	<0.001	2.34 (1.19–4.61)	0.013

Model 1 Adjusted for age, gender. Chronic rhinitis status, drinking and first-degree family history of NPC. Model 2 Further adjusted by smoking status (never/ever). Model 3 Further adjusted by the levels of VCA-lgA (lower/higher). Trace elements were added 10 and transformed by log.

Restricted cubic spline displayed the association between levels of Cd concentration on a continuous scale and NPC risk was J-shaped (*P* for nonlinearity < 0.001); when we compared with the reference category (after transformed;1.004–1.017 μ g/L; 33rd–67th centiles), the multivariable adjusted OR for NPC risks were 2.30 (95%CI, 1.26 to 3.38) for the highest tertile (after transformed; >1.017 μg/L; 68th–100th centiles), 1.09 (95%CI, 0.88 to 2.53) for the lowest tertile (log-transformed; <1.003 μg/L 1st–32nd centiles; Figure 1). And there was a linear relationship between serum Mn and NPC risk (*P* for nonlinearity = 0.519). When the concentration of serum Mn was lower than 8.73 μg/L [log (Mn + 10) = 1.27 μg/L], serum Mn showed a weak protective effect for this cancer, with OR < 1; After exceeding this value, the OR of NPC risk became >1 and increased linearly with the increase of serum Mn concentration.

Stratified analyses by potential effect modifiers (Supplementary Table S4) showed that NPC risk associated with serum Mn and Cd were slightly higher in the ever-smokers and those with high level of VCA-lgA. The risk of NPC associated with the highest tertile of Mn compared with the reference category was 4.41 (95%CI, 2.04 to 9.80) in ever-smokers vs. 3.53 (95%CI, 1.32 to 9.99) in never-smokers, and 2.55 (95%CI, 1.20 to 5.48) in ever-smokers vs. 1.47 (95%CI, 0.54 to 4.05) in never-smokers for Cd. Similarly, the risk of NPC associated with the highest tertile of Mn compared with reference category was 6.28 (95%CI, 1.82 to 11.02) in high antibody level group vs. 5.29 (95%CI, 1.60 to 9.27) in low antibody level group, and 2.43 (95%CI, 1.02 to 6.04) in high antibody level group vs. 1.55 (95%CI, 0.63 to 3.62) in low antibody level group for Cd. We further explored the other risk factors, including chronic rhinitis status, alcohol consumption and first-degree family history of NPC, and the potential synergistic effects of trace elements in the carcinogenesis of nasopharyngeal epithelial cells, and no obvious interactions have been found, with all of the *p* > 0.05.

## 4. Discussion

This multi-center case–control study probed into the relationship between trace elements and NPC risk in Guangdong Province, a region with high NPC incidence. Of the 8 common trace elements investigated, our results showed that serum Mn and Cd were both significantly positive associated with NPC risk in both single-and multiple-element models; and restricted cubic splines showed that there was a linear increasing trend between Mn and NPC risk, while for Cd there was a J-type correlation. But no associations with other trace elements (Cr, Co, Ni, As, Sr, and Mo) were found.

First of all, the reason for studying the relationship between these 8 trace elements and NPC is that all of them play important physiological and metabolic roles in the human body. In addition, previous studies have shown a deficiency or excessive intake of the trace elements of As, Cd, Ni and Sr. had a close ecological relationship with the occurrence of NPC, or some trace elements were closely associated with other types of tumors, such as Cr for lung, thyroid and exocrine pancreatic cancers (22–24), Mn for respiratory cancer and respiratory disease mortality (25), Co for respiratory toxicity (26) and Mo for breast cancer (27). Therefore, we conducted this case–control study for an in-depth exploration of the relationships between these trace elements and NPC. Of the limitation in our laboratory testing ability, we did not test for Zn and Tin, although there have been reported positive associations of NPC or regional liver cancer with these two trace elements (15).

Generally, the main routes of general populations to Cd exposure are water and food intake (1). Although some NPC patients may reduce their dietary intake due to physical discomfort, such as nasal congestion, tinnitus, nasal discharge stained with blood, in theory, this can only reduce the level of serum trace elements in patients with NPC. However, in our study, we still found a positive correlation between higher serum trace elements and NPC risk. Existing studies have reported that the Cd level in the soil of Guangdong Province exceeded Chinese food safety threshold (12), which might result in elevated serum Cd concentration in the local residences (28) and the high prevalence in southern China. Smoking is confirmed as another source of Cd exposure (29), and might also increase the risk of NPC in this population. However, we cannot distinguish the exposure sources for each individual, such as trace elements in the workplace, and a more detailed study should be conducted in the future.

Our result of a moderate positive correlation between Cd and NPC risk in southern China is consistent with another case–control study in Tunisia, an area with low incidence of NPC (18). Our study, however, has a slightly higher OR than that in Tunisia (Average OR = 2.30 and 1.31, respectively), the potential cause of which might be that people in high NPC incidence are more sensitive to Cd exposure, which might be due to the NPC susceptibility genes in NPC endemic regions. Several epidemiologic investigations of genetic susceptibility to NPC have discovered SNPs in relation to genetic polymorphisms in cytochrome P450 (CYP) and glutathione-S-transferase (GST) gene families encode phase I and II xenobiotic metabolism enzymes involved in the biotransformation of chemicals such as the toxin of Cd (30–32). However, more biological mechanisms for the sensitivity of trace element in cancer patients or people in endemic areas are still needed. Cd has been confirmed as the first group of human carcinogen by the International Institute for the Classification of Cancer and closely related to a variety of cancers, including prostate and hormone-related cancer (33). According to etiological studies of other cancers, it can be related to its function in promoting hypermethylation (34–36), malignant transformation and DNA repair inhibition (37).

Mn is an essential trace element for the human body, but excessive intake will also be harmful to human health. In our study, the median concentration of serum Mn in healthy people of Guangdong province (4.52 μg/L) was obviously higher than those in other studies from China (3.30 μg/L) (38) and European countries (2.32 μg/L) (39). This may relate by serious Mn pollution of groundwater in the Pearl River Delta (40). Some studies have shown that Mn in three types of drinking water in Zhaoqing area of Guangdong Province has exceeded the standard, and the over-standard rate of well water is as high as 26.2% (41, 42). In addition to exposure from water, some living habits such as drinking tea (43) and ingesting herbal plants (44) also affect the level of Mn in the human body. Cantonese people have the habit of drinking herbal tea and herbal soup. Early symptoms of NPC are often confused with influenza, prompting patients to consume herbal diets more frequently to manage these symptoms prior to a clinical diagnosis, which may relate with a higher Mn content in NPC patients (45). Although there is no definite evidence for the relationship between Mn levels and tumors, it is speculated that a high Mn level may affect DNA replication and repair courses for cell mutations, DNA damage and chromosome aberrations (46). This analysis is the first to report a strong positive relationship between Mn and NPC risk and a linear-relationship toward higher risk (25, 47). The role of Mn in the NPC development, however, needs additional investigation.

It is worth mentioning that some studies have shown that Ni also correlates with NPC (16). An epidemiological survey found a significantly higher Ni level in rice, drinking water and hair of local inhabitants in high-risk regions than in low counterparts. In addition, in high-risk regions, there was also a higher Ni content in NPC patients than in controls (15). In our study, only single-element models showed a moderate correlation between Ni and NPC. This correlation was not shown in the multiple-element models, which might be because of the insufficient sample size, or Ni was affected by other known or unknown confounding factors. For example, our study found that the correlation coefficients between Ni and Mn, Ni and Cd are 0.363 (*p-*value < 0.001) and 0.278 (*p-*value < 0.001), respectively. However, this does not rule out the possibility that there is no correlation between Ni and NPC, which needed to be studied with a larger sample size.

In the stratified analysis, the NPC risk associated with Mn and Cd were slightly higher in the ever-smokers and the high antibody level group than those in the never-smokers and the low EBV antibody level group. This may be due to the synergistic effect (21, 48, 49) between trace elements and the two risk factors in the carcinogenesis of nasopharyngeal epithelial cells, although the *p* value of interactions were higher than 0.05.

Until now, the cumulative evidence powerfully displays a causal role of EBV in the incident of NPC; However, EBV alone is not an adequate cause of NPC. Environmental cofactors prompt NPC development. It is believed that the accumulation of genome instability induced by environmental factors can facilitate EBV persistent infection in the precancerous nasopharyngeal epithelium. Once infected, EBV latent genes give growth and survival benefits that lead to NPC occurrence (50, 51). According to this etiological model, we postulate that long-term exposure to the trace elements, e.g., Cd (34–36), Ni (52), and Mn (46), might impose various genetic damage or alterations in nasopharyngeal epithelial cells, which further mediates EBV infection and promotes NPC development.

There are several strengths of this study. First of all, the intensity of our research demonstration is high. We comprehensively compared the relationship between eight serum trace elements and NPC with a multi-center sample collection. Strict quality control was applied and the quality of laboratory testing was high. Moreover, our research results are relatively robust. Both single and multiple-element models were utilized and restricted cubic splines were also used to analyze the nonlinear relationship between the concentration of serum trace elements and the risk of NPC. We found a linear increasing trend between Mn and NPC risk, while for Cd there was a J-type correlation.

However, our study has obvious limitations. First of all, our study is based on retrospective data; Secondly, trace elements in serum do not reflect long-term exposure levels; Thirdly, our research lacks occupational exposure information and specific amounts of alcohol consumption and pack years of smoking for the participants; Finally, the results cannot be directly extended to those in the low-risk areas; Consequently, further studies with larger cohorts and systematic clinical evidence are expected to shed light on the causal relationship between them.

## 5. Conclusion

In conclusion, Mn and Cd were positively related with NPC risk. Mn and Cd in certain concentration elevate the risk of NPC. In addition, prospective studies on the relationship between trace elements and NPC should be considered in the future.

## Data availability statement

The data analyzed in this study is subject to the following licenses/restrictions: information that could compromise research participant privacy or consent. Explicit consent to deposit raw data was not obtained from the participants. Requests to access these datasets should be directed to S-MC, caosm@sysucc.org.cn.

## Ethics statement

The studies involving human participants were reviewed and approved by Sun Yat-sen University Cancer Center. The patients/participants provided their written informed consent to participate in this study.

## Author contributions

X-YG, S-MC, and A-HL contributed to conception and design of the study. S-HX and WC organized the database. X-YG, HW, and XL performed the statistical analysis. X-YG wrote the first draft of the manuscript. S-MC, H-NZ, and XY wrote sections of the manuscript. All authors contributed to the article and approved the submitted version.

## Funding

This work was supported by the National Key Research and Development Program of China (grant numbers: 2020YFC1316905 and 2017YFC0907102) and the National Natural Science Foundation of China (grant numbers: 81872700 and 82073625).

## Conflict of interest

The authors declare that the research was conducted in the absence of any commercial or financial relationships that could be construed as a potential conflict of interest.

## Publisher’s note

All claims expressed in this article are solely those of the authors and do not necessarily represent those of their affiliated organizations, or those of the publisher, the editors and the reviewers. Any product that may be evaluated in this article, or claim that may be made by its manufacturer, is not guaranteed or endorsed by the publisher.

## Supplementary material

The Supplementary material for this article can be found online at: https://www.frontiersin.org/articles/10.3389/fnut.2023.1142861/full#supplementary-material

## Abbreviations

As: Arsenic
Cd: Cadmium
CI: Confidence interval
Co: Cobalt
Cr: Chromium
EBV: Epstein–Barr virus
Mo: Molybdenum
Mn: Manganese
Ni: Nickel
NPC: Nasopharyngeal carcinoma
OR: Odds ratio
Sr.: Strontium
